# *Trichuris trichiura* infection in children: Two case reports and literature review

**DOI:** 10.1097/MD.0000000000042114

**Published:** 2025-04-18

**Authors:** Xinxin Chen, Zhiling Wang

**Affiliations:** a Department of Pediatrics, West China Second Hospital, Sichuan University, Chengdu, PR China; b Key Laboratory of Birth Defects and Related Diseases of Women and Children (Sichuan University), Ministry of Education, Chengdu, PR China.

**Keywords:** children, colonoscopy, *Trichuris trichiura*

## Abstract

**Objective:**

To summarize the clinical manifestations, diagnosis and treatment of *Trichuris trichiura* infection in children to increase the awareness of this disease among clinical physicians.

**Methods:**

The clinical data of 2 children infected with *T trichiura* were retrospectively analyzed, and the literature on *T trichiura* infection in Chinese children was reviewed.

**Results:**

Two patients were admitted to the hospital because of “recurrent blood in the stool for more than 9 months,” “a minimum hemoglobin level of 46 g/L” and “anal warts for more than 1 year.” The diagnosis was confirmed in both patients by colonoscopic detection of parasites, and the outcome was favorable after treatment with albendazole. A literature review revealed that the number of whipworm infections reported in Chinese children significantly decreased after 2008; abdominal pain and blood in the stool are the most common gastrointestinal symptoms of whipworm infection in children; and most children experience anemia, developmental delay and malnutrition. During auxiliary examinations, only 0.79% of the children had increased eosinophils in their peripheral blood, and only 3.97% had eggs in their stool. A total of 96.03% of the children were diagnosed by the colonoscopic discovery of parasites.

**Conclusion:**

Despite a significant decrease in incidence in recent years, pediatric whipworm infection cannot be ignored. The clinical manifestations of whipworm infection in children are nonspecific, but few children have eggs in their stool. The detection of parasites with colonoscopy is currently the main diagnostic criterion for whipworm infection, but the indications for microscopic examination should be strictly controlled.

## 1. Introduction

*Trichuris trichiura*, also known as the whipworm, is an intestinal parasite that mainly infects the ileocecal region and causes nonspecific inflammation in the intestinal tract. Between 1988 and 1992, China was 1 of the most affected countries, with the average whipworm infection rate reaching 18.8%.^[1]^ The development of methods for preventing and controlling soil-transmitted nematodes have helped decrease the incidence of whipworm infection each year; in 2020, only 0.16% of the populations of national monitoring points, mainly the mild-climate Yunnan and Hainan, were infected with whipworm.^[2,3]^ As a result, clinicians, especially young doctors, generally lack an understanding of whipworm infection. Coupled with its atypical clinical manifestations and a low detection rate for eggs in the feces, this disease is easily misdiagnosed. Whipworm infection is common in children, even those younger than 2 years of age, with the peak age of infection ranging from 5 to 15 years.^[4,5]^ In addition to digestive tract involvement, children with long-term and repeated infections also develop systemic symptoms such as malnutrition, anemia, slowed growth and potentially developmental delay. Here, we reviewed cases of whipworm infection in 2 children, and along with a literature review, we summarized the epidemiology, clinical manifestations, diagnosis, and treatment of whipworm infection in Chinese children to increase clinicians’ understanding of this disease.

## 2. Case information

### 2.1. Patient 1

A 4-year and 2-month-old boy of Yi nationality, was admitted to the hospital due to “recurrent blood in the stool for more than 9 months with disease exacerbation for 3 days.” For more than 9 months, the patient had black and occasionally bloody loose stools without mucus, 1 to 4 times/day, paroxysmal abdominal pain, and no fever. Three days prior, the patient passed a large amount of blood in the stool, accompanied by a pale complexion, dizziness, and fatigue. Routine blood tests revealed that the hemoglobin (Hb) level was 46 g/L. The patient had not gained weight since he fell ill and had a long history of drinking unboiled water. His growth and development had been normal before illness.

Physical examination revealed pale, no skin rash, no abnormalities in the heart or lungs, a soft abdomen, and no tenderness, rebound tenderness, or muscle tension; however, the liver and spleen were not palpable under the ribs, there were no palpable masses, and the bowel rhythm was 3 to 5 beats/min. No external hemorrhoids and anal fissure were noted.

Laboratory tests: Routine blood tests revealed the following: WBC 6.8 × 10^9^/L, NEUT% 44.4%, EOS% 6.7%, EOS 0.46 × 10^9^/L, Hb 46g/L, MCV 62.8 fL, MCH 15.3 pg, MCHC 243.0 g/L, PLT356 × 10^9^/L, CRP < 0.5 mg/L, and reticulocyte% 2.14%. Stool analysis revealed the following: WBC 5 to 10/high power field (HPF), RBC ++++/HPF, OB (+), no eggs, and negative parasite antibodies. No abnormalities were identified in liver or kidney function, electrolytes, or coagulation function, and the results of Meckel diverticulum scan were not abnormal.

**Figure 1.**
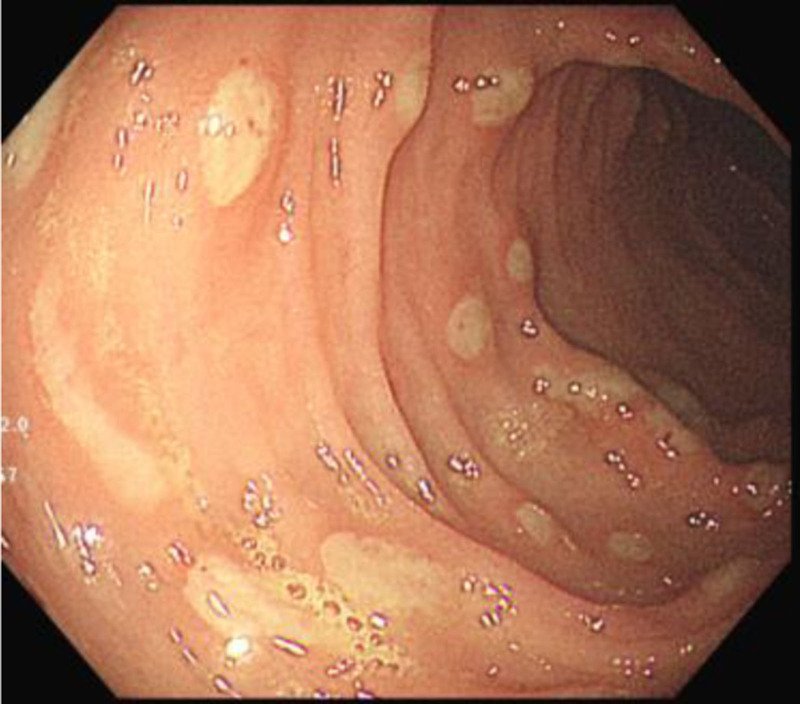
Multiple superficial ulcers in the colon.

**Figure 2.**
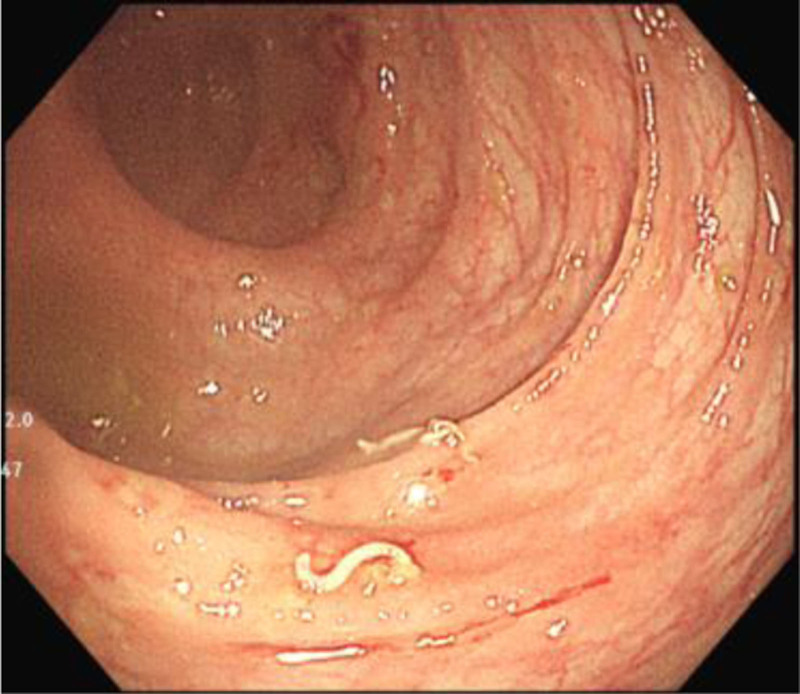
Parasites observed in the ascending colon.

**Figure 3.**
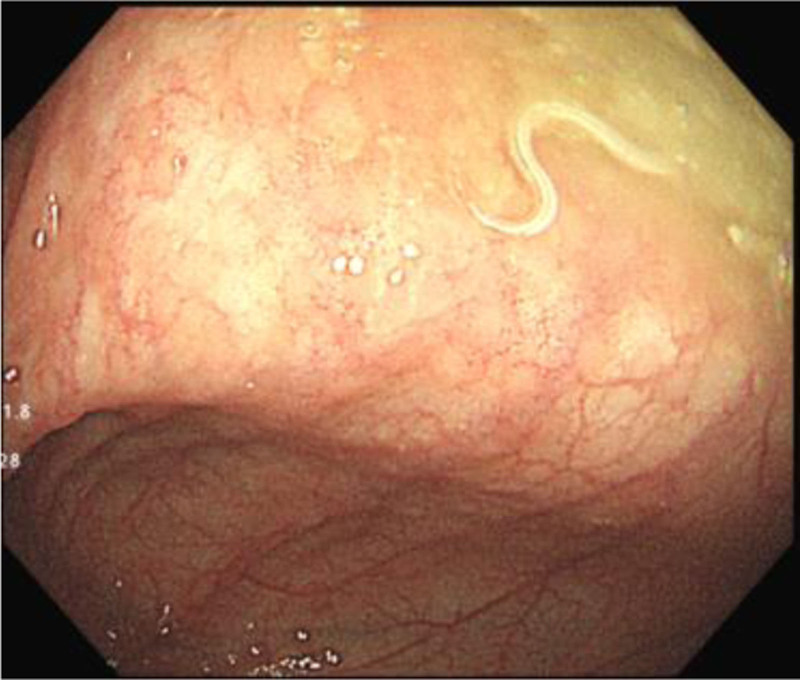
Parasites observed in the ileocecal region.

Diagnosis and treatment: Microcytic hypochromic severe anemia was diagnosed according to Clinical presentation and Laboratory tests. Lower gastrointestinal bleeding was considered because of the bloody stools. Analyzing the causes of bleeding, hemorrhoids and anal fissure were excluded based on physical examination. Despite the elevated WBC in stool, infectious enteritis wasn’t prioritized and antibiotic wasn’t used due to the normal CRR and bloody stool without mucus. Stool culture was further performed and was negative. Meckel diverticulum was suspected, Meckel scan with 99m technetium pertechnetate was performed but revealed no significant findings. Abdominal CT revealed sigmoid colon tortuosity with focal wall thickening and indistinct ileocecal region. Upper and lower endoscopy were further done and revealed scattered irregular superficial ulcers approximately 0.3 to 0.5 cm in size on the ileocecal valve and the mucosa of the cecum, colon, and rectum (Fig. 1) and 3 white, striped, worm-like substances in the ascending colon and hepatic flexure (Fig. 2). Peristalsis was observed with the head end embedded in the mucosa. An intestinal parasitic infection was initially suspected. The anterior segments of the parasites were slightly elongated, the posterior ends were thick, and the caudal ends were curved ventrally in a circular fashion. The parasite laboratory confirmed an infection of whipworm, and the patient was diagnosed with trichuriasis, multiple ulcers in the colon, and hemorrhagic anemia (severe).

The patient received albendazole (200 mg, bid, 3 days), achieving complete hematochezia resolution prior to discharge with progressive symptom attenuation. During the whole treatment, the patient kept a good compliance without complaint except for transient periumbilical pain requiring no medical intervention. Within half a month after discharge from the hospital, the patient had occasional bloody eyes in the stool but had otherwise recovered. During the 2-year follow-up, the patient’s growth and development were satisfactory, no additional anemia was observed, and the bloody and mucinous stools had disappeared.

### 2.2. Patient 2

A 10-year-old boy of Yi nationality, was admitted to the hospital due to “anal warts for more than 1 year.” One year prior, the parents reported pink warts at the anus orifice during bowel movements, and sometimes returned spontaneously after defecation. The patient produced stools 1 to 2 times a day, with occasional blood streaks, no mucus, and no anal pain; the patient was never brought for medical treatment. The child tended to drink unboiled water but had grown and developed normally.

Physical examination revealed no rash on the skin, and the heart, lungs, and abdomen examinations were normal. The perianal skin was intact, and no anal fissures, perianal abscesses, external hemorrhoids, fistulas, or rectal prolapse were found.

Laboratory tests: Leukocyte, Eosinophil count, Red blood cell counts, liver or kidney function, electrolytes, and coagulation function were all within normal limits. Stool examination revealed the following: WBC 3 to 5/HPF, RBC 5 to 10/HPF, and OB (+).

Diagnosis and treatment: Due to the complaint of anal warts, colonoscopy was prioritized and revealed a slender white parasite moving in the ileocecal region (Fig. 3) and measuring approximately 1.5 cm in length. Scattered ulcers could be observed in the ascending colon, transverse colon, and descending colon, and dense punctate deposits could be observed in the sigmoid colon and rectum. After removal, the parasite presented with a slender anterior end of approximately 2 cm length, and part of it was pinched off, resembling a horsewhip. Examination confirmed that it was a whipworm (female). The patient presented with concurrent trichuriasis and rectal mucosal prolapse. Albendazole therapy (200 mg, bid, 3 days) normalized stool consistency but failed to resolve persistent post-defecation anal protrusion. Full treatment adherence was documented with no adverse events observed. During the 2-year follow-up, the patient experienced occasional prolapse during bowel movement, but his stool characteristics were normal, and his height and weight developed normally.

Unfortunately, neither patient had a follow-up colonoscopy as prescribed.

## 3. Literature search and review

The China National Knowledge Infrastructure, Wanfang, VIP, PubMed, and Embase databases and the Cochrane Library were searched using the keywords “Trichuris trichiura,” “soil-transmitted nematode” and “children.” The retrieval time was set from database establishment to December 2023. A total of 5 studies comprising 124 pediatric cases were included.^[6–10]^ The results of the literature search for trichuriasis in Chinese children are summarized in Table 1.

**Table 1:** Summary of information on trichuriasis in Chinese children.

Authors, publication date	Number of cases	Age	Sex	Environment and residence	Hygiene and eating habits	Clinical manifestations	Important auxiliary examinations	Diagnostic basis	Treatment and outcome
Liu et al, 1996^[6]^	4	9 to 13	Male (2) and female (2)	※	Eating uncooked vegetables, drinking unboiled water, and not washing hands before meals and after using the toilet	Abdominal pain, diarrhea, weight loss	Routine stool test: whipworm eggs found	Eggs in stool	Mebendazole or albendazole
Liu et al, 2000^[7]^	117	11 mo to 14 yr	Male (76) and female (41)	Urban (16 cases), rural (101 cases)	※	All had blood in the stool, intermittent diarrhoea, abdominal pain, and some mild anemia	Routine stool tests: RBC (++ to +++), WBC (+ to +++), OB (+), no eggs; colonoscopy: whipworms found mostly in the left colon	Adult parasites found under colonoscopy	Mebendazole: blood in the stool disappeared after the 1-mo follow-up visit. No whipworm was found in 18 patients during follow-up colonoscopy. The intestinal mucosal injury healed.
Chen et al, 2018^[8]^	1	4 yr and 4 mo	Female	Rural area	Liked to walk barefoot	Diarrhea, blood in the stool, tenesmus, perianal pruritus, weight loss	Routine stool test: RBC +++/HPF, OB (+); Modified Kato thick smear test: whipworm eggs found; Colonoscopy: parasites found in the ileocecal region	Parasite eggs found in the stool; adult parasites found under colonoscopy	Albendazole: normal stool at the follow-up 2 and 4 wk after discharge.
Mu et al, 2019^[9]^	1	15 yr	Male	※	※	Abdominal pain, syncope, anemia, and tenderness in lower right abdomen	Routine blood test: Hb 98 g/L; Colonoscopy: whipworms found in the ileocecal region	Adult parasites found under colonoscopy	Levamisole: the abdominal pain improved 3 d later, and the patient was discharged from the hospital.
Li et al, 2022^[10]^	1	2 yr	Male	Rural area	Occasionally played in dirt	Diarrhea, blood in the stool, slowed growth	Routine blood test: EOS% 7.9%, EOS 1 × 10^9^/L; routine stool test: OB (+); colonoscopy: multiple parasites observed under colonoscopy, mainly in the ascending colon	Adult parasites found under colonoscopy	Albendazole
Patient 1*	1	4 yr and 2 mo	Male	Rural area	Drank unboiled water	Blood in the stool, abdominal pain, dizziness, fatigue, anemia, and no weight gain	Routine blood test: Hb 46 g/L; Routine stool test: OB (+), RBC ++++/HPF; colonoscopy: parasites observed in the ascending colon and hepatic flexure	Adult parasites found under colonoscopy	Albendazole: the patient’s growth and development was satisfactory during the 2-yr follow-up period. The patient had no anemia, and the stool conformation was normal.
Patient 2*	1	10 yr	Male	Rural area	Drank unboiled water	Anal warts; recurrent diarrhea between 1 and 5 yr of age	Routine stool test: RBC 5 to 10/HPF, OB (+); colonoscopy: worms found in the ileocecal region	Adult parasites found under colonoscopy	Albendazole: during the 2-yr follow-up period, the stool characteristics were normal, and the patient’s growth and development was satisfactory.

※:represents not mentioned.

EOS = eosinophil, Hb = hemoglobin, HPF = high power field, OB = occult blood, RBC = red blood cells, WBC = white blood cells.

* Unpublished.

## 4. Discussion

*T trichiura*, also known as whipworm, is a soil-transmitted nematode that, together with hookworm and the Ascaris worm, is located distribution but is found mainly in warm, humid tropical and subtropical developing countries with poor economic and sanitation conditions. Whipworms once widely infected much of the population in China. With improvements in sanitation conditions, increasing health awareness, and the widespread implementation of measures such as “four reforms and 1 deworming,” the infection rate for children in China has decreased significantly.^[2,3]^ This trend was also indirectly reflected in our search of published literature on whipworm infections in Chinese children. Whipworms are transmitted mainly through the fecal–oral route, with humans being the only source and host of infection, the prevalence of which is largely affected by hygienic conditions. The review of the literature revealed that most infected children were from rural areas (82.04%) or areas primarily populated by ethnic minorities, which may be related to the relatively weak economy, poor sanitation conditions and poor hygiene habits therein. Therefore, improving integrated management in rural and ethnic minority areas is important for controlling infections of soil-transmitted nematodes, such as whipworm, in China.

Adult whipworms mainly parasitize the cecum, but in severe cases, they can also infect the colon, rectum, and terminal ileum. The front end of the parasites burrows into the intestinal epithelium, feeds on blood and tissue fluid, and causes disease through mechanical damage, chemical stimulation and nutrient plundering in the host intestine. The clinical symptoms and course of the disease, the number of parasites and the response to treatment are closely related to the degree of intestinal mucosal injury. The incidence of whipworm infection is particularly high among children, and the literature review revealed that the average age of infection was 5.4 years. The most common symptoms are abdominal pain, anemia and blood in the stool, the later of which is mainly caused by mechanical damage to the intestinal mucosa by the parasites. According to the literature, the amount of bleeding caused by each adult parasite is 0.005 mL/d.^[4]^ Therefore, the greater the number of parasitic worms and the longer the course of the disease are, the more severe the anemia is. Most children with whipworm disease as well as blood in the stool present with mild to moderate small-cell hypochromic anemia, while severe anemia can be observed in certain cases. The Hb level in the case report by Zanwar^[11]^ and of case 1 in this study were only 38 g/L and 46 g/L, respectively. Stimulation of the intestinal mucosal nerve plexus by the parasites is thought to play a role in the symptoms of abdominal pain, diarrhea, nausea, and vomiting. Because the cecum is the most common site of involvement, children with whipworm infection tend to present with pain and tenderness in the right lower abdomen, and so this infection needs to be differentiated from appendicitis. In addition, complications of whipworm infection, such as appendicitis, intestinal obstruction, and intussusception, are also related to cecal involvement. Fortunately, the incidence of these complications is low.

Systemic symptoms are another common manifestation of whipworm infection in children. Even among children with mild or no obvious gastrointestinal symptoms, misdiagnosis is common. In addition to anemia, long-term heavy whipworm infection can lead to developmental delay or malnutrition and even impairment of mental development and cognitive function in children^[4,6,8,10]^; studies have shown that the degree of impairment is associated with the course of the disease and the number of parasites.^[12]^ Even after the parasite has been eradicated, the effects of infection on height may persist into adulthood. One patient who experienced long-term infection in childhood was only 160 cm tall at the age of 26, significantly lower than the height of his elder brother (180 cm).^[13]^

Notably, Chen^[8]^ reported a case in which children were coinfected with whipworm and ascarids, which suggests that immunocompromised children may host a variety of parasites, especially those with a consistent epidemic distribution, potentially resulting in more complicated and severe symptoms.

Auxiliary examinations of whipworm infection include the eosinophil count and stool examinations for eggs. A literature review revealed that only 0.79% of children with whipworm infection had an elevated eosinophil count in the peripheral blood; therefore, a normal eosinophil count cannot rule out whipworm infection. The detection of whipworm eggs in the stool is the gold standard for the diagnosis of infection, but these eggs are small and therefore easy to miss.^[14]^ A literature review suggested that the percentage of eggs found in the stool was only 3.97%. Only 1 such case was reported in the literature after 2008, which may be related to a significant reduction in the number of parasites compared with previous cases. In this case, the direct saline smear method was also negative for eggs, whereas the modified Kato method was positive. Due to the higher detection rate of eggs, the modified Kato method is recommended by the World Health Organization for screening parasites.^[4]^

Another gold standard for the diagnosis of whipworm disease is the discovery of adult whipworms and/or the findings of mucosal pathology revealing whipworms or eggs under colonoscopy. At present, the confirmed cases reported in the literature essentially relied on colonoscopy. In addition to parasites in different parts of the colon, the morphology of the intestinal mucosa can also be observed under a microscope, which, combined with mucosal pathology, can provide an important basis for the diagnosis and differential diagnosis of the disease. Under colonoscopy, whipworms can be found in the cecum or other intestinal segments, with the front end deeply buried in the mucosa. After the whipworms are removed, they appear with a light gay coloration and a shape like a horsewhip; the anterior 3/5 of the thread is a thin thread, and the posterior 2/5 is as thick as a whip handle. Female whipworms are 35 to 50 mm long, with blunt and rounded ends; males are 30 to 45 mm long, with tails curled toward the ventral surface in a ring shape.^[14]^ Among the reported cases of whipworm infection confirmed by colonoscopy domestically and from foreign institutions, many were unintentionally discovered due to gastrointestinal symptoms or health examinations.^[15–20]^ These findings indicate that a whipworm infection may not show symptoms or that the symptoms lack specificity. Therefore, we may have underestimated the current rate of whipworm infection. The key to reducing missed diagnoses is improving our diagnostic awareness and understanding of this disease. The findings from our cases and the literature reflect the importance of colonoscopy in the diagnosis of trichuriasis. Nevertheless, due to the invasiveness of colonoscopy, the indications should be strictly controlled.

At present, the treatment strategy for trichuriasis involves implementation of large-scale preventive deworming in endemic areas and therapeutic deworming for sporadic cases. Commonly used anthelmintic drugs include mebendazole, albendazole, oxantel, and pyrimidine compounds. Although the results of the present study revealed that the administered drugs have good deworming effects and that the recurrence rate was low, due to the presence of whipworms with their front ends embedded in the intestinal tract, the treatment efficacy of drugs against whipworms reported in the literature is worse than that of drugs against roundworms and hookworms.^[4]^ The negative conversion rate of stool worm eggs is only approximately 70%, but endoscopic spraying of anthelmintic agents and forceps removal of parasites provide new methods for treatment.^[20]^

This study highlights 2 clinical cases from Yi ethnic rural communities, in which delayed medical intervention contributed to disease progression. Diagnostic challenges persist due to limited healthcare accessibility, caregiver awareness gaps, and inconsistent clinical expertise, often leading to misdiagnosis and aggravated morbidity. Therefore, targeted health education, increased medical investment, and economic support in these areas should be the focus of prevention and control strategies for whipworm infection in the future. Clinicians evaluating children with persistent gastrointestinal symptoms (e.g., hematochezia, abdominal pain) or systemic manifestations (e.g., anemia, growth retardation) should maintain a suspicion for trichuriasis, even when the initial stool parasite test is negative. Colonoscopy is recommended for definitive diagnosis in clinically suspicious cases, as demonstrated by the 2 patients in this study who tested negative for ova initially but were later confirmed via endoscopic examination. Empirical anthelminthic therapy may be considered when endoscopic confirmation is unavailable, though posttreatment monitoring should incorporate laboratory follow-up rather than relying solely on symptomatic improvement.

There are some limitations in our study. For both cases, neither had a clear epidemiological history, and both should be followed up with colonoscopy to evaluate for mocosal damage, but not in either case because their guardians refused.

## 5. Conclusion

*Trichuris trichiura* infection in children still cannot be ignored, especially in rural and ethnic minority areas with underdeveloped economies and poor sanitation conditions. Clinical presentations of *Trichuris trichiura* infection range from asymptomatic to life-threatening anemia and colonoscopy should be done if suspected.

## Acknowledgments

The authors have no funding and conflicts of interest to disclose and thank the parents of the children involved in this study.

## Author contributions

**Writing – original draft:** Xinxin Chen.

**Writing – review & editing:** Zhiling Wang.
